# Cotton Pectate Lyase GhPEL48_Dt Promotes Fiber Initiation Mediated by Histone Acetylation

**DOI:** 10.3390/plants13172356

**Published:** 2024-08-23

**Authors:** Anlin Zhong, Xianyan Zou, Zhenzhen Wei, Lei Gan, Jun Peng, Yonghui Li, Zhi Wang, Yuanyuan Liu

**Affiliations:** 1 Zhengzhou Research Base, State Key Laboratory of Cotton Bio-Breeding and Integrated Utilization, Zhengzhou University, Zhengzhou 450001, China; 2 State Key Laboratory of Cotton Bio-Breeding and Integrated Utilization, Institute of Cotton Research, Chinese Academy of Agricultural Sciences, Anyang 455000, China; 3 Center for Yunnan Plateau Biological Resources Protection and Utilization, College of Biological Resource and Food Engineering, Qujing Normal University, Qujing 655011, China; 4 National Nanfan Research Institute (Sanya), Chinese Academy of Agricultural Sciences, Sanya 572024, China

**Keywords:** pectate lyase, fiber initiation and elongation, trichome, histone acetylation

## Abstract

*GhPEL48_Dt*, a Pectate lyase (PEL, EC4.2.2.2), is a crucial enzyme involved in cell-wall modification and pectin degradation. Studies have shown that the *GhPEL48_Dt* also plays a significant role in cotton-fiber development; however, the specific function and regulatory mechanism of *GhPEL48_Dt* in cotton-fiber development are still not fully understood. Here, we found that the histone deacetylase inhibitor-Trichostatin A significantly reduces the transcript levels of *GhPEL48_Dt* and its enzyme activity. Further, silencing of *GhPEL48_Dt* significantly inhibits the initiation and elongation of cotton fibers by promoting pectin degradation, and the heterologous expression of GhPEL48_Dt promotes the development of trichomes and root hairs in Arabidopsis, which suggests that *GhPEL48_Dt* plays a positive and conserved role in single cell i.e., fiber, root hair, and leaf trichome development. Collectively, this paper provides a comprehensive analysis of the fundamental characteristics and functions of GhPEL48_Dt in fiber development, including the regulatory role of histone acetylation on GhPEL48_Dt, which contributes to the understanding of pectin degradation pathways and establishes a theoretical foundation for elucidating its regulatory mechanism.

## 1. Introduction

Cotton, a member of the genus *Gossypium* in the Malvaceae family, is highly valued in the textile industry due to its natural fiber derived from seed epidermis. Cotton fiber is an excellent raw material for the textile industry and exhibits huge economic value. The quality and quantity of cotton fiber directly affect the quality and production costs of textiles, efficient land utilization, and farmers’ income. Therefore, studying cotton-fiber formation and creating superior-quality cotton germplasms are necessary.

At present, a lot of work on fiber development has been reported from various perspectives such as phytohormones, molecular regulation, and structure characterization [1]. For instance, gibberellic acid (GA) is crucial in stimulating cotton-fiber initiation and elongation by affecting the expression levels of *GhGA20ox*, a pivotal gene catalyzing bioactive GA synthesis [2]. Similarly, cotton-fiber growth is regulated by the actin cytoskeleton and microtubule organization using live-cell imaging analysis, and is promoted by cytoskeleton-related gene *GhACTIN1* [3,4,5].

Cotton-fiber development is a programmed and orderly progress that can be divided into four acknowledged processes, including fiber initiation, elongation (primary cell-wall formation), secondary cell-wall (SCW) formation, and maturation [1]. The primary cell wall (PCW) is composed of cellulose fibrils, proteins, and polysaccharides. Pectin, the important component of polysaccharides, exists in an esterified or de-esterified form in PCW. Pectate lyase (PEL) is an enzyme responsible for homogalacturonan depolymerization by breaking up the α-1,4 glycosidic bond of homogalacturonan, accompanied by the generation of 4,5-unsaturated oligosaccharides [6,7]. PEL contributes to loosening cell walls, exerting a vital role in pollen development [8], fruit softening [9], and maturation [10,11], and thickening secondary cell walls [12]. In the Gossypium genus, *PEL* gene family has been well documented and 53, 42, 83 *PEL* genes were identified in *G. raimondii*, *G. arboretum*, and *G. hirsutum*, distinctly [13]. The *PEL* gene family is classified into five subfamilies and subfamily V is preferentially expressed in fiber [13].

Histone acetylation, a reversible posttranslational modification in lysine or N-terminal, participates in the regulation of gene transcription, cell division, DNA damage repairs, and so on [14]. Histone acetyltransferases (HATs) and histone deacetylases (HDACs) play a vital role in sustaining the balance of lysine acetylation to histone acetylation [15]. Previous research has indicated that the use of trichostatin A (TSA), a histone deacetylase inhibitor, can hinder the initiation and development of cotton fiber [16]. *GhPEL48_Dt* belonging to PEL subfamily V has higher expression levels in cotton fiber of long-fiber cotton cultivars than that of short-fiber cotton cultivars [6]. Previously, enzyme activity analysis indicates *GhPEL48_Dt* encodes a pectate lyase with cleavage activity on de-esterified pectin and can positively regulate cotton-fiber elongation [6]. However, the regulation mechanism of *GhPEL48_Dt* on fiber development remains unclear. In this study, we conducted molecular analyses and revealed that the transcript level of *GhPEL48_Dt* (Gh_D03G0971) decreased significantly in cotton ovules treated with 10 µM TSA and that it also involved in the fiber initiation and elongation in cotton and the development of trichomes and root hairs in arabidopsis, which unveiled that *GhPEL48_Dt* regulates fiber initiation by histone acetylation.

## 2. Results

### 2.1. Gene Transcript Level and Protein Enzyme Activity of GhPEL48_Dt Were Regulated by TSA

**Figure 1:**
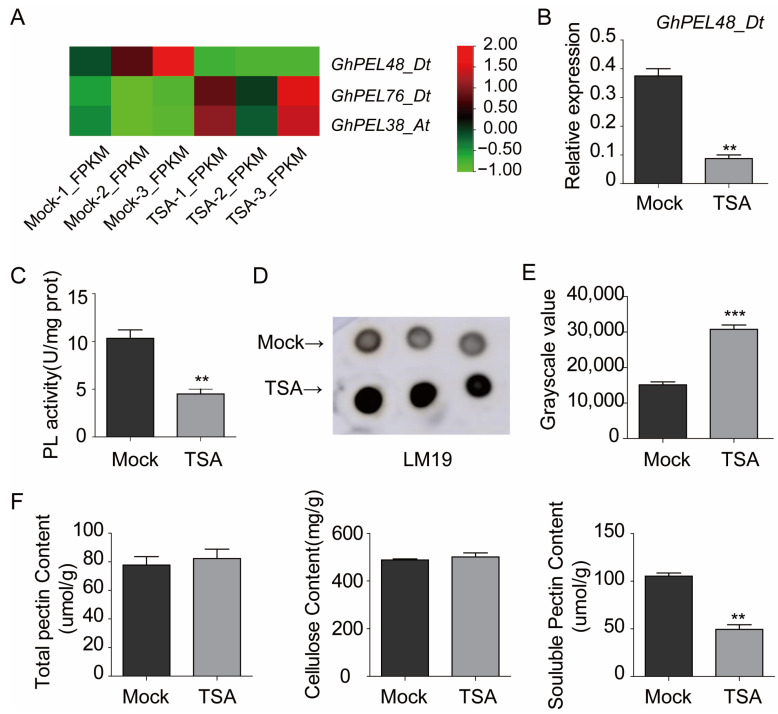
The gene transcript level and protein enzyme activity analysis of GhPEL48_Dt after exogenous TSA application. (**A**) The transcript level analysis of *GhPEL48_Dt*, *GhPEL76_Dt*, and *GhPEL38_At* in the TSA-treated ovules. (**B**) Relative expression levels of *GhPEL48_Dt* in TSA-treated ovules and control. (**C**) Pectate lyase activity in TSA-treated ovules and untreated ovule. (**D**) De-esterified pectin contents in TSA-treated ovules and control through dot immunobinding assay. Three dots in each row represent three experimental replicates. (**E**) Gray scale value statistics of immune signals were presented in the form of a bar graph. (**F**) The content analysis of total pectin, cellulose, and soluble pectin in TSA-treated and untreated ovules. Error bars represent ± standard error (SE) of triplicate experiments, and asterisks indicate the level of significance between the mean values determined by student’s *t*-test (** *p* ˂ 0.05, *** *p* ˂ 0.01).

We analyzed the transcript levels of *GhPEL48_Dt* based on publicly available TSA-treated ovule transcriptome data [16], and the result showed *GhPEL48_Dt* was down-regulated by histone acetylation during the fiber initiation stage (Figure 1A). Furthermore, qRT-PCR analysis showed the mRNA levels of *GhPEL48_Dt* exhibited a 3-fold decrease in cotton treated with 10 µM TSA (Figure 1B), which is consistent with the transcriptome data treated by TSA, indicating that histone acetylation plays a negative role in the transcription of *GhPEL48_Dt*. Further, the protein enzyme activity of GhPEL48_Dt was down-regulated by TSA (Figure 1C). Additionally, we used LM19 antibody to label de-esterified pectin in the ovules treated with TSA in the immune spot assay and found that the content of de-esterified pectin significantly accumulated in the ovules treated with TSA (Figure 1D,E). To further analyze the effect of TSA on the cell wall, we monitored cell wall component contents in ovules treated with TSA. The results showed that the total pectin and cellulose content in the TSA-treated ovule material did not show a significant difference, but the soluble pectin content was significantly reduced compared with the control group (Figure 1F). These results suggest that PEL enzyme activity may be regulated by histone acetylation, leading to the cell wall component change and affecting fiber development.

### 2.2. Structure Characteristics, Expression Patterns and Subcellular Localization Analysis of GhPEL48_Dt

**Figure 2:**
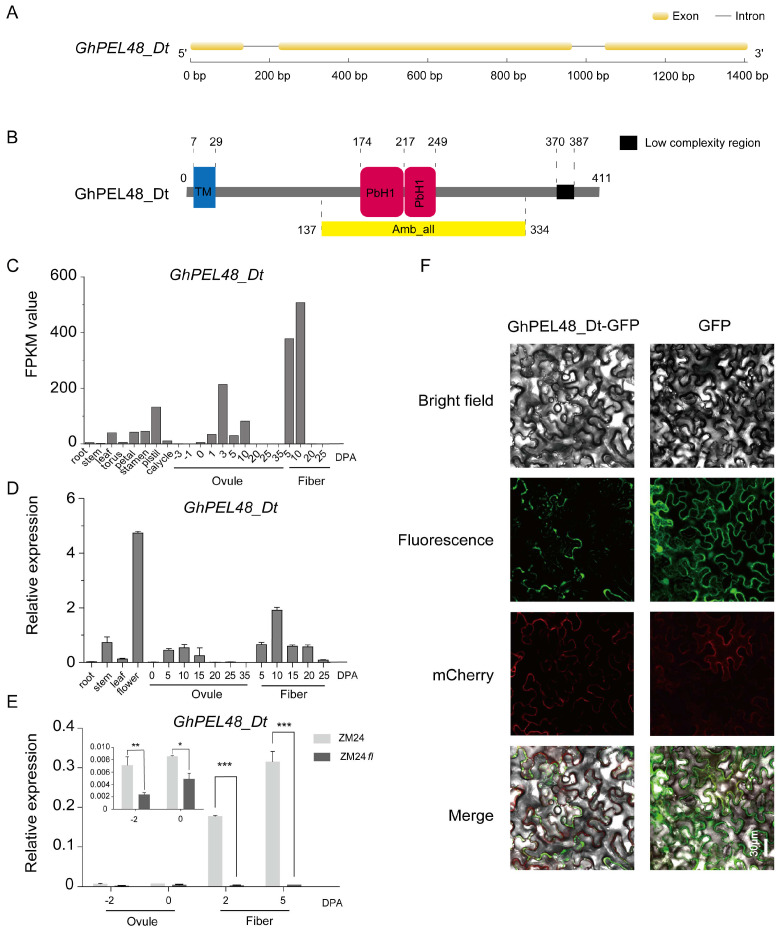
Structure characteristics, expression patterns, and subcellular localization analysis of GhPEL48_Dt. (**A**) The gene structure of *GhPEL48_Dt.* Yellow boxes indicate exons, black lines indicate introns. (**B**) Conserved domain analysis of GhPEL48_Dt protein. Blue box represents the transmembrane domain, yellow box represents Amb_all domains (containing two PbH1 domains), and black box represents the low complexity structure. (**C**) *GhPEL48_Dt* expression levels in different tissues of cotton based on the transcriptome sequencing data. DPA, day post anthesis. (**D**) Relative transcript levels of *GhPEL48_Dt* in different tissues of cotton were analyzed by quantitative real-time PCR. The *UBQ7* gene was used as a control. Three independent replicates were conducted. (**E**) GhPEL48_Dt expression analysis in ZM24 and *ZM24fl* (is a glabrous mutant). Error bars represent the standard error (SE) of triplicate experiments. Asterisks indicate a significant difference (Student′s *t*; * *p* < 0.05, ** *p* < 0.01, *** *p* < 0.001) (**F**) Subcellular localization of GhPEL48_Dt in *N. benthamiana* by laser scanning confocal microscopy.

To dissect the role of GhPEL48_Dt in fiber development, we extracted the gene sequence from the genome data of *Gossypium hirsutum*. The gene annotation showed that the CDS sequence of *GhPEL48_Dt* contains 1236 bp and encodes 411 amino acids. The gene structure is composed of three exons and two introns (Figure 2A). Protein structure prediction contributes to exploring the gene function. Therefore, we analyzed the sequence characteristics of GhPEL48_Dt according to the online web of SMART (Simple Modular Architecture Research Tool) and predicted that GhPEL48_Dt protein mainly contains a transmembrane domain and two PbH1 domains, which may endow GhPEL48_Dt with the function of catalyzing the polysaccharide (Figure 2B).

To explore the possible functions of *GhPEL48_Dt* in cotton, we detected the expression levels of *GhPEL48_Dt* in different tissues and developmental stages of fiber and ovule. The results showed that the expression levels of *GhPEL48_Dt* were higher during 5–20 DPA, which hints that GhPEL48_Dt may play an important role in elongation (Figure 2C,D). In addition, *GhPEL48_Dt* also exhibited high expression levels in floral organs (Figure 2C,D). At the same time, the expression level of *GhPEL48_Dt* was significantly down-regulated in ZM24fl, the nearly-isogenic fibreless mutant of ZM24, compared to ZM24, which further illustrates the importance of *GhPEL48_Dt* in fiber development (Figure 2E). To explore the subcellular localization of GhPEL48_Dt, the transient expression of a binary vector pCAMBIA2300-GhPEL48_Dt-GFP in *Nicotiana benthamiana* leaves was analyzed. Green fluorescent signals were detected near mCherry, a red fluorescent plasma membrane marker by confocal laser scanning microscopy (Figure 2F), which indicates that GhPEL48_Dt may be a membrane protein. To determine whether PEL48 is located on the cell membrane, we conducted subcellular location analysis using an efficient PEG-mediated transient gene expression system in tobacco protoplasts. No signal was detected in plasma member (Figure S1), which indicates that PEL48 is not on the cell membrane and may be localized in the extracellular region near plasma member.

### 2.3. GhPEL48_Dt Promotes Fiber Initiation and Elongation

**Figure 3:**
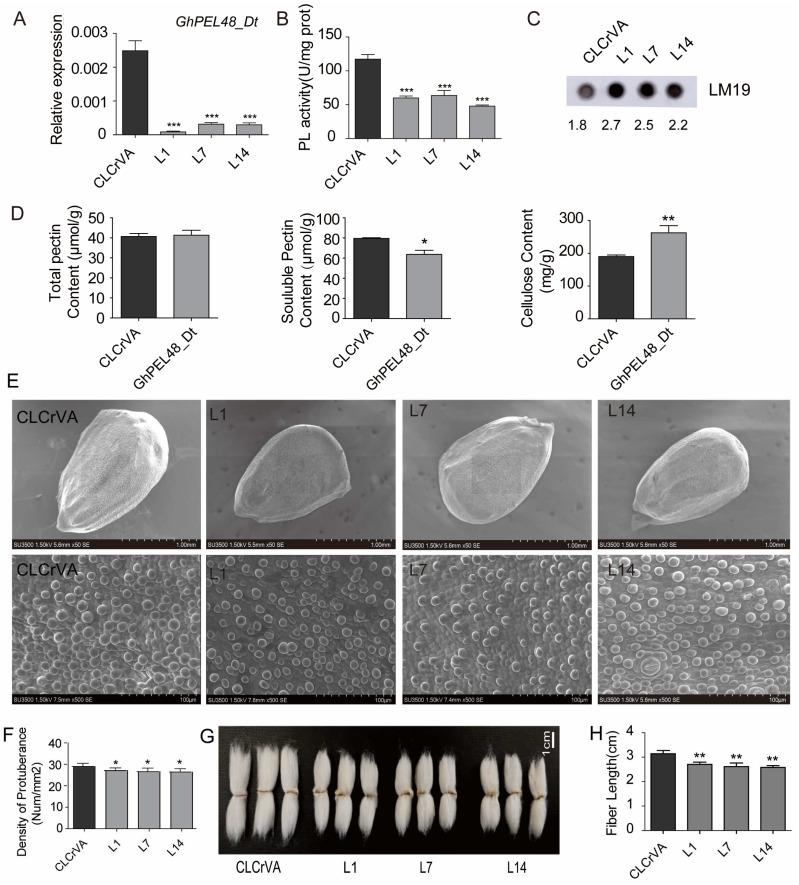
Functional analysis of GhPEL48_Dt in fiber development. (**A**) Relative expression level of *GhPEL48_Dt* in the ovule of silencing GhPEL48_Dt lines and control (CLCrVA). (**B**) Pectate lyase enzyme assays in the ovule of GhPEL48_Dt silencing lines and control. (**C**) De-esterified pectin content analysis in the ovule of GhPEL48_Dt silencing lines and control by dot immunobinding assay. LM19 antibody was used to label the de-esterified pectin. The numbers below the image represent the grayscale values of the immune signals. (**D**) The content analysis of total pectin, cellulose, and soluble pectin in GhPEL48_Dt silencing lines ovule and control. (**E**) The phenotypes of fiber initiation in the ovule silencing GhPEL48_Dt and control. (**F**) Analysis of initial fiber number in the lines silencing GhPEL48_Dt and control. The raw data of initial fiber number are provided in Table S1. (**G**) The phenotype of mature fibers in silencing GhPEL48_Dt lines and control. (**H**) Analysis of mature fiber length in silencing GhPEL48_Dt and control. Data are presented as the mean ± SE of three independent experiments. Statistical significance was determined using one-way ANOVA combined with Tukey’s test. * *p* < 0.05; ** *p* < 0.01; *** *p* < 0.001.

To further investigate the biological function of GhPEL48_Dt in cotton-fiber development, we used VIGS technology to obtain *GhPEL48_Dt* silencing plants. qRT-PCR analysis showed significantly down-regulated transcript level of *GhPEL48_Dt* in L1, L7, and L14 VIGS lines (Figure 3A). Moreover, enzymatic assay indicated the activity of PEL was significantly reduced in the *GhPEL48_Dt* silencing lines (Figure 3B). Besides, immunoblot analysis using LM19 antibody labeled with de-esterified pectin, a substrate of pectinase, showed that the immune signal in silencing *GhPEL48_Dt* ovule was significantly higher than in the control group. Figure 3C suggests that reduced pectinase activity of L1, L7, and L14 leads to the accumulation of their substrate, de-esterified pectin. These data indicate that *GhPEL48_Dt* can boost notably the degradation of the de-esterified pectin.

To examine the effect of *GhPEL48_Dt* on the changes in cell wall components, we detected the total pectin, soluble pectin, and cellulose content in the ovule. The results showed the total pectin content did not show a significant difference in *GhPEL48_Dt* silenced plants and the control group, but the *GhPEL48_Dt* silenced plants contained lower soluble pectin content and higher cellulose content compared with the control group (Figure 3D). The results verify the positive role of *GhPEL48_Dt* in splitting pectin.

In addition, the density of fiber initials in the silenced plants at 0 DPA was lower than that in the control group (Figure 3E,F). Moreover, the mature fiber length of the *GhPEL48_Dt* silenced plants was significantly shorter (Figure 3G,H). These results indicate that *GhPEL48_Dt* can facilitate pectin degradation and produce more soluble pectin to boost cell-wall loosening, resulting in fiber development.

### 2.4. GhPEL48_Dt Promotes the Growth of Epidermal Hairs, and Root Hairs in Arabidopsis thaliana

**Figure 4:**
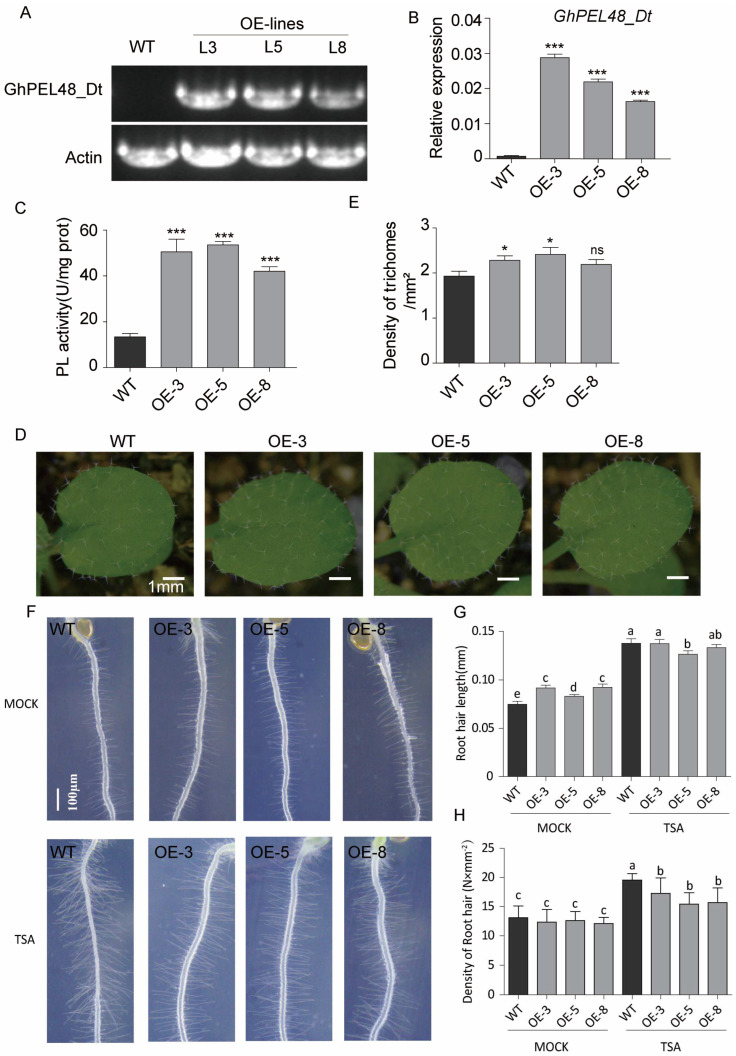
Generation and functional analysis of *GhPEL48_Dt* transgenic Arabidopsis. (**A**) Semi-quantitative PCR of *GhPEL48_Dt* transgenic Arabidopsis. (**B**) Relative expression levels of *GhPEL48_Dt* in wild-type and Arabidopsis overexpressing *GhPEL48_Dt.* (**C**) Pectate lyase activity of wild-type and GhPEL48_Dt overexpressing Arabidopsis. (**D**) The phenotype of trichomes in wild-type and *GhPEL48_Dt* transgenic Arabidopsis. (**E**) Statistical analysis of trichome density in wild-type and *GhPEL48_Dt* transgenic Arabidopsis. (**F**) The phenotypes of root hair in TSA-treated and untreated wild-type and GhPEL48_Dt transgenic Arabidopsis. (**G**) Statistical analysis of root hair length in *GhPEL48_Dt* transgenic Arabidopsis, TSA-treated and control. (**H**) Quantitative analysis of root hair density in *GhPEL48_Dt* transgenic Arabidopsis, TSA-treated and control. Gray box represents control; black box represents lines treated with TSA. Asterisks in figureB, C and E indicate a significant difference (Student′s *t*; ns *p* > 0.05, * *p* < 0.05, *** *p* < 0.001). Letters above the bars (in (**G**,**H**)) indicate signifcant diference according to one-way ANOVA analysis at *p* < 0.05.

To further verify the role of GhPEL48_Dt in trichome development, the heterologous overexpression of GhPEL48_Dt in *Arabidopsis thaliana* was performed. By PCR amplification with specific primers, specific stripes were detected in three *GhPEL48_Dt* overexpressing transgenic Arabidopsis plants (Line 3, 5, 8) (Figure 4A). Furthermore, qPCR analysis showed the transcript levels of *GhPEL48_Dt* significantly increased in the three transgenic lines (Figure 4B). The enzymatic assay of GhPEL48_Dt showed that the pectate lyase activity increased in Arabidopsis overexpressing GhPEL48_Dt lines (Figure 4C).

To determine the change in pectate lyase activity effect, the plant growth, root, and trichome development of the transgenic Arabidopsis were observed and analyzed. The results indicated that overexpression of GhPEL48_Dt in *Arabidopsis thaliana* led to an increase in trichome density on the leaf epidermis (Figure 4D,E). However, the root hair length of the transgenic Arabidopsis was longer, but the density of the root hair was not significantly changed compared with the wild type (Figure 4F). These results suggest that the increased PEL activity of GhPEL48_Dt contributes to trichome development in Arabidopsis. To analyze the impact of histone acetylation on *GhPEL48_Dt,* we examined the root hair changes in transgenic Arabidopsis plants expressing *GhPEL48_Dt* by applying TSA. The exogenous TSA application showed significantly reduced root hair length and density in lines expressing *GhPEL48_Dt* compared to the wild type (Figure 4F–H), suggesting that *GhPEL48_Dt* is regulated by histone acetylation.

## 3. Discussion

Cotton fiber, the primary product of cotton, has significant economic value as the main raw material for the textile industry. Producing high yield and superior quality fibers has always been the goal of cotton breeding. To date, research on cotton-fiber initiation and development has been reported with multiple aspects such as molecular regulation, structure development, etc. [3]. To be specific, MYB-bHLH-WDR (MBW) is an important transcription factor complex involved in the regulation of fiber development [17]. Secondary cell-wall (SCW) thickening is an important stage affecting fiber quality development and *CesA4*, *7*, and *8* are dominant genes involved in this stage [18]. Epigenetic modifications (DNA methylation, histone modification, and non-coding RNA-mediated post-transcriptional regulation) are important for gene expression regulation [19,20,21,22]. To date, researchers have focused on the role of DNA methylation and other histone modifications in fiber development; however, studies on histone acetylation are still scarce.

PEL, EC4.2.2.2 is involved in cell-wall modification by cleaving α-1,4-glycosidic linkages in esterified pectin. PEL also plays essential roles in many developmental processes, such as flower organ formation and fruit ripening and softening [23,24]. Recently, *PEL* genes have been identified and analyzed preliminarily in cotton. Previous studies have shown that *GhPEL48_Dt* is highly expressed during fiber development and in long-fiber cotton cultivars [6]. Furthermore, earlier transcriptome data revealed a significant downregulation of *GhPEL48_Dt* expression level under TSA treatment [16]. However, there are still no comprehensive reviews to summarize the characteristics and function of GhPEL48_Dt and the related mechanism of PEL regulating cotton-fiber development. Therefore, we speculated that *GhPEL48_Dt* is regulated by histone acetylation to participate in fiber development.

In this study, we systematically documented the gene structure and function of *GhPEL48_Dt,* which refined the basic characteristics of *GhPEL48_Dt*. In addition, we found that silencing of *GhPEL48_Dt* resulted in the decrease in PEL activity, soluble pectin, and the increase in cellulose content in the cotton ovule, inhibiting fiber formation and elongation indicating the positive roles of PEL in cotton-fiber development by affecting pectin cleaving and cell-wall modification. Also, *GhPEL48_Dt* promotes epidermal hair and root hair development of Arabidopsis, which manifests that *GhPEL48_Dt* plays a conserved role in promoting trichome development. It can be seen that the function of GhPEL48_Dt in modifying plant cell-wall structure is conservative, all achieved by degrading pectin to increase the elasticity and flexibility of the cell wall, thereby promoting plant growth and the development of floral organs or trichomes, and so on.

Although there have been reports on the important functions of GhPEL48_Dt as a pectinase [6], the regulatory mechanism upstream of *GhPEL48_Dt* remains unclear and has been poorly studied. This paper preliminary elucidates the regulation mechanism of histone acetylation on *GhPEL48_Dt* from transcript level by the application of TSA. Furthermore, GhPEL48_Dt transgenic *Arabidopsis* with TSA treatment affected the regulation of root hairs and trichomes, indicating that the function of GhPEL48_Dt is regulated by histone acetylation.

Currently, the function of PEL has been widely studied in various plants, but regulatory mechanisms remain unclear. This paper may provide a reference for the regulatory mechanisms of PEL in other plants. The genes and complexes involved in histone acetylation modification of *GhPEL48_Dt* remain unknown and need to be further analyzed. In addition, transcription factors can directly bind to the gene promoter and finely regulate the expression level of genes, influencing the functions and characteristics of cells and participating in various growth and developmental processes of organisms and adaptation to the changing environment [25,26,27]. Currently, there is limited understanding of the upstream transcription factors of *GhPEL48_Dt*, and further analysis is required. The upstream of *GhPEL48_Dt* may involve multiple factors working together to regulate its expression, such as GCN5-ADA2b, TFs, and so on, thereby influencing pectin metabolism and fiber development. Therefore, extensive research is still needed to elucidate the complex regulatory mechanisms of *PEL48*.

## 4. Materials and Methods

### 4.1. Plant Materials and Growth Conditions

*Arabidopsis thaliana* ecotype Columbia-0 (Col-0) seeds were sterilized three times with 10% NaClO and rinsed twice with distilled water. Then, sterilized seeds were placed on 1/2 MS medium with agar for germination in a growth chamber at 22 °C, 70% humidity, and under 16 h light/8 h dark conditions. Seeds of tobacco (*Nicotiana benthamiana*) and *Gossypium hirsutum* (CRI) (ZM24) were sown in 90 mm × 65 mm plastic pots containing organic soil with perlite and grown in greenhouses at 70% humidity, 30 °C, and 16 h/8 h light/dark.

### 4.2. Plasmid Construction

The full-length coding DNA sequence of *GhPEL48_Dt* was extracted from ZM24 genome and primers were designed using Primer Premier 5 software. The primers are listed in Table S2. To generate overexpression lines, a 1236 bp CDS fragment of *GhPEL48_Dt* was cloned behind a CaMV 35S promoter of pCAMBIA2300 vector containing a flag tag. The CDS sequence was also inserted into the pCAMBIA2300 vector containing a GFP tag for fluorescence observation. The specific~300 bp fragments of GhPEL48_Dt were amplified and cloned into pCLCrVA for VIGS.

### 4.3. Generation of Transgenic Arabidopsis Plants

The 35S:GhPEL48_Dt recombinant plasmids, together with Agrobacterium strain GV3101, was introduced into *Arabidopsis* plants using the floral dip method. A 1/2 MS medium containing 50 mg L^−1^ kanamycin was used to screen transgenic plants, and specific PCR primers were designed to identify transgenic plants (Table S2).

### 4.4. Virus-Induced Gene Silencing of GhPEL48_Dt in Cotton

A virus-induced gene silencing (VIGS) method was used to silence the expression of GhPEL48_Dt in cotton. First, the plasmid of pCLCrV-GhPEL48_Dt and pCLCrVA were transformed into LBA4404. Then, *Agrobacterium* containing pCLCrV-GhPEL48_Dt and pCLCrVA were mixed separately with pCLCrVB *Agrobacterium,* and were injected into the cotyledons of two-week-old cotton seedlings, respectively. The plants were kept in dark for 48 h, and then transferred to the long-day condition (16 h light/8 h dark).

### 4.5. Subcellular Localization Analysis of GhPEL48_Dt

The recombinant plasmids pCAMBIA2300-GhPEL48_Dt-GFP were transformed into *A. tumefaciens* strain GV3101 and cultured in 50 mL YEB medium for 48 h in a shaker (180 rpm) at 28 °C. Then, the supernatant was discarded, bacteria were collected by centrifuging at 6000 rpm for 6 min, and the pellet was resuspended with infiltration buffer (10 mM MgCl_2_·6H_2_O, 0.5 M MES, and acetosyringone) until OD 600 reached 1.0. The suspension was inoculated into 25-day-old tobacco leaves using a needle-free syringe (1 mL) incubated for 24 h in dark and then transferred to the long-day condition (16 h light/8 h dark). After 4 days of inoculation, the leaves were cut for fluorescence observation using confocal laser scanning microscopy (Olympus, FV 1200, Tokyo, Japan) under 488 nm laser excitation.

### 4.6. In Vitro Culture and SEM Analysis

Flowers were collected at −1 days post anthesis (DPA) and ovaries were sterilized with 75% alcohol. Ovaries were dissected in a sterile environment, and intact ovules were carefully stripped from the ovaries and immediately cultured in a 6-well culture plate containing 5 mL BT medium without TSA (Millipore, 647925, Burlington, MA, USA) or with 10 µM TSA in a plant growth chamber at 30 °C in the dark. Then, SEM (Hitachi, SU3500, Tokyo, Japan) was used to observe the fiber initiation and development. The fiber counts were measured and analyzed by ImageJ2 software.

### 4.7. Gene Expression Analysis

Total RNA was extracted from plant tissues using Qiagen RNeasy kit and RNA aqueous small-scale phenol-free total RNA isolation kit (Ambion, Austin, TX, USA) according to the manufacturer’s instructions. A total of 2 µg RNA was used to generate first-strand cDNA with 1 µL oligo (dT) using the SuperScript RT-PCR System (Invitrogen, Waltham, MA, USA). A total of 2 µL cDNA, 10 µL QPCR Mix, 0.6 µL forward and reverse primers, and 6.8 µL RNA-free water were mixed. qRT-PCR was performed according to the previous procedure [28]. Three independent experiments were performed as biological replicates. *UBIQUITIN 7* and *Actin* were used as internal reference genes in cotton and Arabidopsis to normalize all samples, respectively. The 2^−ΔΔCT^ method was used to calculate the relative expression level.

### 4.8. Probing Dot Blots with LM19 Antibodies

The assay was essentially performed as previously described [29], with minor modifications. Briefly, pectin was extracted from coarse cell walls using CDTA buffer consisting of 25 mM Tris-HCl, 50 mM CDTA, 25 mM DTT, and 1.5% (*w*/*v*) PVPP. A total of 1 µL diluted pectin solutions were spotted onto nitrocellulose membranes, then the membranes were dried and incubated with LM19 antibody. Tanon-5200 chemiluminescent imaging system (Tanon, Shanghai, China) was applied to visualize the image.

### 4.9. Detection of Cell Wall Component Contents

A total pectin assay kit (BC1405, Solarbio, Beijing, China) is used to determine total pectin contents according to the manufacturer’s protocol. To be specific, the tissue samples were extracted with extract buffer homogenate in 90 °C water bath twice for 30 min each time. The precipitation was retained after centrifugation. Extract solution 2 was added, hydrolyzed at 90 °C for 1 h, and the supernatant was taken to be measured. After the supernatant was mixed with the reaction system, the absorbance at 530 nm was measured at 25 °C for 30 min.

A soluble pectin content determination kit (BC4125, Solarbio, China) was used to determine the content of soluble pectin. A total of 1 g cotton ovule was ground and cleaned alternately with extract solution I and acetone at 90 °C to obtain precipitate (coarse cell wall). Extract solution II was added to the precipitate and soaked for 2 h to obtain crude cell wall, then extraction solution III was added and the mixture was centrifuged three times to acquire the supernatant. The supernatant was mixed with the reaction solution and reacted at 25 °C for 30 min. The absorbance at 530 nm was determined.

The cellulose content was determined using the cellulose content determination kit (BC4285, Solarbio, China). Cellulose was extracted by adding concentrated sulfuric acid to crude cell wall. The supernatant was mixed with the reaction solution and reacted at 95 °C for 10 min, then the absorbance at 620 nm was determined. The standard curves were made with the standard solution and the cell wall component contents were calculated according to the formula in the manual.

### 4.10. Pectate Lyase Activity Assay

Pectate lyase activity was detected according to the protocol described in the manual of pectin lyase kit (BC2645, Solarbio, China). The plant samples were grounded and dissolved in the extract solution, then the mixture was centrifuged and supernatant was kept. Reaction solution was added to supernatant and reacted at 40 °C for 30 min, then the absorbance at 235 nm was determined.

### 4.11. Statistical Analysis

SPSS statistics software (version 17.1) was employed to analyze the experimental data by one-way analysis of variance or Student’s *t*-test.

## 5. Conclusions

*GhPEL48_Dt* promotes fiber initiation and elongation by degrading pectin and relaxing cell walls. Besides, *GhPEL48_Dt* also enhances the development of trichomes and root hairs in Arabidopsis. These results indicate *GhPEL48_Dt* plays a positive conserved role in single-cell fiber and trichome development. This study provides a theoretical basis for further elucidation of molecular regulation mechanism of GhPEL48_Dt in fiber development.

## Data Availability

The data that support the findings of this study are available on request from the corresponding author.

## Supplementary Materials

The following supporting information can be downloaded at: https://www.mdpi.com/article/10.3390/plants13172356/s1, Figure S1: Subcellular localization of GhPEL48_Dt in transiently transfected tobacco protoplasts; Table S1: The total number of protuberance under 500× scanning electron microscopy; Table S2: Primers used in this study.

## References

[1] Wen X., Chen Z., Yang Z., Wang M., Jin S., Wang G., Zhang L., Wang L., Li J., Saeed S. (2023). A comprehensive overview of cotton genomics, biotechnology and molecular biological studies. Sci. China Life Sci..

[2] Xiao Y.H., Li D.M., Yin M.H., Li X.B., Zhang M., Wang Y.J., Dong J., Zhao J., Luo M., Luo X.-Y. (2010). Gibberellin 20-oxidase promotes initiation and elongation of cotton fibers by regulating gibberellin synthesis. J. Plant Physiol..

[3] Huang G., Huang J.-Q., Chen X.-Y., Zhu Y.-X. (2021). Recent advances and future perspectives in cotton research. Annu. Rev. Plant Biol..

[4] Li X.B., Fan X.P., Wang X.L., Cai L., Yang W.C. (2005). The cotton *ACTIN*_1_ gene is functionally expressed in fibers and participates in fiber elongation. Plant Cell.

[5] Yu Y., Wu S., Nowak J., Wang G., Han L., Feng Z., Mendrinna A., Ma Y., Wang H., Zhang X. (2019). Live-cell imaging of the cytoskeleton in elongating cotton fibres. Nat. Plants.

[6] Wang H., Guo Y., Lv F., Zhu H., Wu S., Jiang Y., Li F., Zhou B., Guo W., Zhang T. (2010). The essential role of *GhPEL* gene, encoding a pectate lyase, in cell wall loosening by depolymerization of the de-esterified pectin during fiber elongation in cotton. Plant Mol. Biol..

[7] Sun H., Hao P., Gu L., Cheng S., Wang H., Wu A., Ma L., Wei H., Yu S. (2020). Pectate lyase-like gene *GhPEL76* regulates organ elongation in *Arabidopsis* and fiber elongation in cotton. Plant Sci..

[8] Jiang J., Yao L., Yu Y., Lv M., Miao Y., Cao J. (2014). *PECTATE LYASE-LIKE10* is associated with pollen wall development in *Brassica campestris*. J. Integr. Plant Biol..

[9] Yang L., Huang W., Xiong F., Xian Z., Su D., Ren M., Li Z. (2017). Silencing of *SlPL*, which encodes a pectate lyase in tomato, confers enhanced fruit firmness, prolonged shelf-life and reduced susceptibility to grey mould. Plant Biotechnol. J..

[10] Jiménez-Bermudez S., Redondo-Nevado J., Munoz-Blanco J., Caballero J.L., López-Aranda J.M., Valpuesta V., Pliego-Alfaro F., Quesada M.A., Mercado J.A. (2002). Manipulation of strawberry fruit softening by antisense expression of a pectate lyase gene. Plant Physiol..

[11] Payasi A., Sanwal G.G. (2003). Pectate lyase activity during ripening of banana fruit. Phytochemistry.

[12] Bai Y., Wu D., Liu F., Li Y., Chen P., Lu M., Zheng B. (2017). Characterization and functional analysis of the poplar *Pectate Lyase-Like* gene *PtPL1-18* reveal its role in the development of vascular tissues. Front. Plant Sci..

[13] Sun H., Hao P., Ma Q., Zhang M., Qin Y., Wei H., Su J., Wang H., Gu L., Wang N. (2018). Genome-wide identification and expression analyses of the pectate lyase (*PEL*) gene family in cotton (*Gossypium hirsutum* L.). BMC Genom..

[14] Narita T., Weinert B.T., Choudhary C. (2019). Functions and mechanisms of non-histone protein acetylation. Nat. Rev. Mol. Cell Biol..

[15] Bajpai S.K., Nisha P.S., Bahadur A., Verma P.C. (2024). Recent advancements in the role of histone acetylation dynamics to improve stress responses in plants. Mol. Biol. Rep..

[16] Wei Z., Li Y., Ali F., Wang Y., Liu J., Yang Z., Wang Z., Xing Y., Li F. (2022). Transcriptomic analysis reveals the key role of histone deacetylation via mediating different phytohormone signalings in fiber initiation of cotton. Cell Biosci..

[17] Huang J., Guo Y., Sun Q., Zeng W., Li J., Li X., Xu W. (2019). Genome-wide identification of R2R3-MYB transcription factors regulating secondary cell wall thickening in cotton fiber development. Plant Cell Physiol..

[18] Huang J., Chen F., Guo Y., Gan X., Yang M., Zeng W., Persson S., Li J., Xu W. (2021). GhMYB7 promotes secondary wall cellulose deposition in cotton fibres by regulating *GhCesA* gene expression through three distinct *cis*-elements. New Phytol..

[19] Gibney E.R., Nolan C.M. (2010). Epigenetics and gene expression. Heredity.

[20] Hamilton J.P. (2011). Epigenetics: Principles and practice. Dig. Dis..

[21] Gagnidze K., Pfaff D.W. (2022). Epigenetic mechanisms: DNA methylation and histone protein modification. Neuroscience in the 21st Century: From Basic to Clinical.

[22] Retis-Resendiz A.M., González-García I.N., León-Juárez M., Camacho-Arroyo I., Cerbón M., Vázquez-Martínez E.R. (2021). The role of epigenetic mechanisms in the regulation of gene expression in the cyclical endometrium. Clin. Epigenetics.

[23] Zhang W.W., Zhao S.Q., Gu S., Cao X.Y., Zhang Y., Niu J.F., Liu L., Li A.-R., Li A.-R., Li A.-R. (2022). FvWRKY48 binds to the pectate lyase *FvPLA* promoter to control fruit softening in *Fragaria vesca*. Plant Physiol..

[24] Mollet J.-C., Leroux C., Dardelle F., Lehner A. (2013). Cell wall composition, biosynthesis and remodeling during pollen tube growth. Plants.

[25] Weidemüller P., Kholmatov M., Petsalaki E., Zaugg J.B. (2021). Transcription factors: Bridge between cell signaling and gene regulation. Proteomics.

[26] He H., Yang M., Li S., Zhang G., Ding Z., Zhang L., Shi G., Li Y. (2023). Mechanisms and biotechnological applications of transcription factors. Synth. Syst. Biotechnol..

[27] Spitz F., Furlong E.E.M. (2012). Transcription factors: From enhancer binding to developmental control. Nat. Rev. Genet..

[28] Zou X., Ali F., Jin S., Li F., Wang Z. (2022). RNA-Seq with a novel glabrous-ZM24 *fl* reveals some key lncRNAs and the associated targets in fiber initiation of cotton. BMC Plant Biol..

[29] Bethke G., Glazebrook J. (2019). Measuring pectin properties to track cell wall alterations during plant–pathogen interactions. Plant Innate Immun. Methods Protoc..

