# Supplementary material for: Cotton Pectate Lyase GhPEL48_Dt Promotes Fiber Initiation Mediated by Histone Acetylation
Source: Plants (Basel). 2024 Aug 23;13(17):2356. doi: 10.3390/plants13172356 (PMC11397362; doi:10.3390/plants13172356)
Supplement: Supplementary file 1 [file plants-13-02356-s001.zip › plants-3065264-supplementary/Supplementary Materials Information.pdf]

Figure S1: Subcellular localization of GhPEL48_Dt in transiently transfected tobacco protoplasts.
Table S1: The total number of protuberance under 500× scanning electron microscopy.
Table S2: Primers used in this study.
