# Supplementary material for: Cotton Pectate Lyase GhPEL48_Dt Promotes Fiber Initiation Mediated by Histone Acetylation
Source: Plants (Basel). 2024 Aug 23;13(17):2356. doi: 10.3390/plants13172356 (PMC11397362; doi:10.3390/plants13172356)
Supplement: Supplementary file 1 [file plants-13-02356-s001.zip › plants-3065264-supplementary/Supplementary Table S2.pdf]

Table S2. Primers used in this study

Primers	Sequences（5′ to 3′ ）
qUBQ7-F	AGAGGTCGAGTCTTCGGACA
qUBQ7-R	GCTTGATCTTCTTGGGCTTG
qGhPEL48_Dt-F	CTGTGGAACCGAAACCCCATT
qGhPEL48_Dt-R	GGGGTTAACGGGGTCATCATCG
qβ-actin-F	TGGTGTCAGGTTGGGATGG
qβ-actin-R	CGTGAGAAGAACAGGGTGCT
GhPEL48_Dt-cYFP-F	GACGCCGGCGGATCCATGGCAAGGACAATGGCA
GhPEL48_Dt-cYFP R	CAGGTCGACTCTAGAACAACGTGAACCTTTCTT
GhADA2b-nYFP-F	GGTACCCGGGGATCCATGGGTCGTTCTCGCGGG
GhADA2b-nYFP-R	GCCGTCGACTCTAGATCATGGCGCAGCAATCCC
GhPEL48_Dt –I AD-F	GGAGGCCAGTGAATTCATGGCAAGGACAATGGCAAT
GhPEL48_Dt –I AD-R	CGAGCTCGATGGATCCATACTGAACGGTAATGCATG
GhPEL48_Dt –II AD –F	GGAGGCCAGTGAATTCATGGCAAGGACAATGGCAAT
GhPEL48_Dt –II AD –R	CGAGCTCGATGGATCCACCGAAGATGGAAACACCAT
GhPEL48_Dt –III AD –F	GGAGGCCAGTGAATTCATGGGTAGCCATGTTTGGGTTGA
GhPEL48_Dt –III AD –R	CGAGCTCGATGGATCCACAACGTGAACCTTTCTTGC
GhPEL48_Dt –IV AD –F	GGAGGCCAGTGAATTCATGGCAAGGACAATGGCAAT
GhPEL48_Dt –IV AD –R	CGAGCTCGATGGATCCGTTGGAAATGGTGATAGCGG
GhPEL48_Dt –V AD –F	GGAGGCCAGTGAATTCATGGTGACCAACATTATCATTCA
GhPEL48_Dt –V AD –R	CGAGCTCGATGGATCCCCGCTATCACCATTTCCAAC
GhPEL48_Dt –VI AD F	GGAGGCCAGTGAATTCATGATCCCTTCACTTCCTCTTCT
GhPEL48_Dt –VI AD R	CGAGCTCGATGGATCCGAGAAGTGGGAGTAGAAGCA
GhPEL48_Dt –VII AD F	GGAGGCCAGTGAATTCATGATTTCCTCTTCCCCTGTTCA
GhPEL48_Dt –VII AD R	CGAGCTCGATGGATCCATACTGAACGGTAATGCATG
GhPEL48_Dt –VIII AD F	GGAGGCCAGTGAATTCATGAATTACATGACTCACCATGA
GhPEL48_Dt –VIII AD R	CGAGCTCGATGGATCCACAACGTGAACCTTTCTTGC
GhPEL48_Dt -AD F	GGAGGCCAGTGAATTCATGGCAAGGACAATGGCAAT
GhPEL48_Dt -AD R	CGAGCTCGATGGATCCACAACGTGAACCTTTCTTGC
GhPEL48_Dt -VIGS-F	TGCCTGCAGACTAGTGTGACCCCAACTGGGAGACT
GhPEL48_Dt -VIGS-R	TAGACCTAGGGGCGCGCCCGGTAATGCATGGACCCCCTG
GhPEL48_Dt cLUC- F	GCGTCCCGGGGCGGTACCATGGCAAGGACAATGGCAAT
GhPEL48_Dt cLUC- R	AAAGCTCTGCAGGTCGACTCAACAACGTGAACCTTTCTTG
GhADA2b nLUC- F	GGGGACGAGCTCGGTACCATGGGTCGTTCTCGCGGGAA
GhADA2b nLUC- R	GTACGAGATCTGGTCGACTGGCGCAGCAATCCCCTTCTTC

Primers	Sequences（5' to 3' ）
GhPEL48_Dt -GFP-F	ATTTGGAGAGGACAGGGTACCATGGCAAGGACAATGGCAAT
GhPEL48_Dt -GFP-R	AGCTCCTCCTCCTCCTCTAGAACAACGTGAACCTTTCTTGC
GhPEL48_Dt -flag-F	CGGGGGACTCTTGAGGATCCATGGCAAGGACAATGGCA
GhPEL48_Dt -flag-R	GTAGTCAGGCGCGCCACAACGTGAACCTTTCTT
