# Supplementary figures and images for: Cotton Pectate Lyase GhPEL48_Dt Promotes Fiber Initiation Mediated by Histone Acetylation
Source: Plants (Basel). 2024 Aug 23;13(17):2356. doi: 10.3390/plants13172356 (PMC11397362; doi:10.3390/plants13172356)

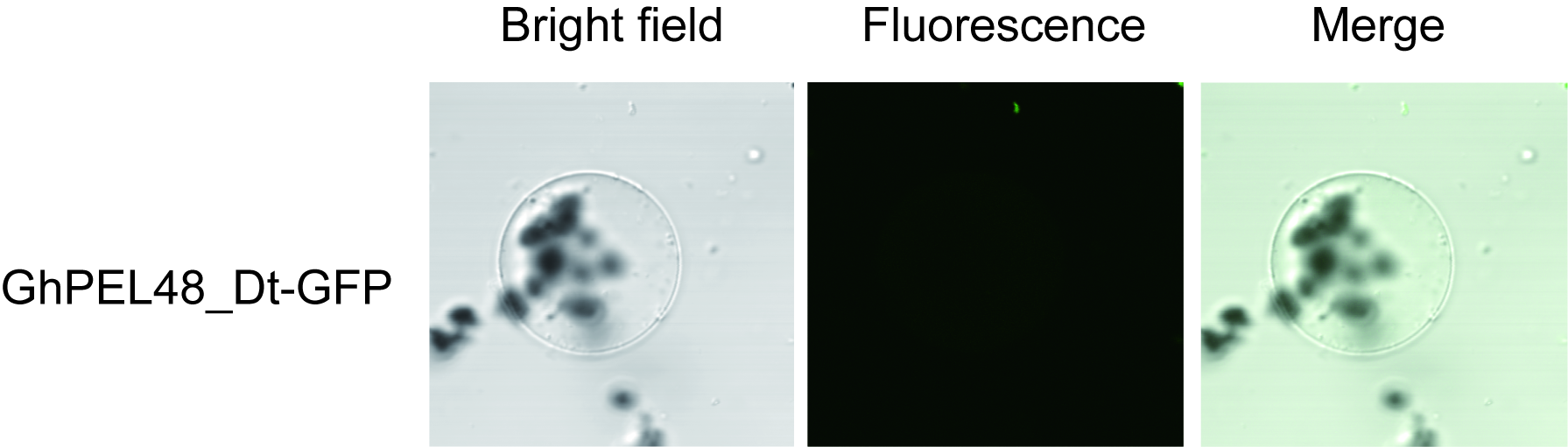

Supplement: Supplementary file 1 [file plants-13-02356-s001.zip › plants-3065264-supplementary/Supplementary Figure S1.tif]
